# Urinalysis findings and urinary kidney injury biomarker concentrations

**DOI:** 10.1186/s12882-017-0629-z

**Published:** 2017-07-06

**Authors:** Girish N. Nadkarni, Steven G. Coca, Allison Meisner, Shanti Patel, Kathleen F. Kerr, Uptal D. Patel, Jay L. Koyner, Amit X. Garg, Heather Thiessen Philbrook, Charles L. Edelstein, Michael Shlipak, Joe El-Khoury, Chirag R. Parikh, on behalf of the TRIBE-AKI Consortium Investigators, Chirag R. Parikh, Jai Raman, Valluvan Jeevanandam, Shahab Akhter, Charles Edelstein, Cary Passik, Judy Nagy, Madhav Swaminathan, Michael Chu, Martin Goldbach, Lin Ruo Guo, Neil McKenzie, Mary Lee Myers, Richard Novick, Mac Quantz, Michael Zappitelli, Ana Palijan, Michael Dewar, Umer Darr, Sabet Hashim, John Elefteriades, Arnar Geirsson, Susan Garwood, Isabel Butrymowicz, Harlan Krumholz, Stephanie Dixon

**Affiliations:** 1 Division of Nephrology, Department of Medicine, Icahn School of Medicine at Mount Sinai, One Gustave Levy Place, Box 1243, New York, NY 10029 USA; 2 Department of Biostatistics, University of Washington, Seattle, WA USA; 3 Division of Nephrology, Department of Medicine, Duke University, Durham, NC USA; 4 Division of Nephrology, Department of Medicine, University of Chicago, Pritzker School of Medicine, Chicago, IL USA; 5 Division of Nephrology, Department of Medicine, Western University, London, ON Canada; 6 Program of Applied Translational Research, Department of Internal Medicine, Yale University School of Medicine, New Haven, CT USA; 7 Division of Nephrology, Department of Medicine, University of Colorado, Denver, CO USA; 8 Division of General Internal Medicine, San Francisco VA Medical Center, University of California, San Francisco, USA; 9 Department of Laboratory Medicine, Yale University School of Medicine, New Haven, CT USA; 10 Division of Nephrology, Department of Medicine, Yale University School of Medicine, New Haven, CT USA

**Keywords:** Biomarkers, Urinalysis, Acute kidney injury, Urine dipstick, Variability

## Abstract

**Introduction:**

Urinary biomarkers of kidney injury are presumed to reflect renal tubular damage. However, their concentrations may be influenced by other factors, such as hematuria or pyuria. We sought to examine what non-injury related urinalysis factors are associated with urinary biomarker levels.

**Methods:**

We examined 714 adults who underwent cardiac surgery in the TRIBE-AKI cohort that did not experience post-operative clinical AKI (patients with serum creatinine change of ≥ 20% were excluded). We examined the association between urinalysis findings and the pre- and first post-operative urinary concentrations of 4 urinary biomarkers: neutrophil gelatinase-associated lipocalin (NGAL), interleukin-18 (IL-18), kidney injury molecule-1 (KIM-1), and liver fatty acid binding protein (L-FABP).

**Results:**

The presence of leukocyte esterase and nitrites on urinalysis was associated with increased urinary NGAL (R^2^ 0.16, p < 0.001 and R^2^ 0.07, p < 0.001, respectively) in pre-operative samples. Hematuria was associated with increased levels of all 4 biomarkers, with a much stronger association seen in post-operative samples (R^2^ between 0.02 and 0.21). Dipstick proteinuria concentrations correlated with levels of all 4 urinary biomarkers in pre-operative and post-operative samples (R^2^ between 0.113 and 0.194 in pre-operative and between 0.122 and 0.322 in post-operative samples). Adjusting the AUC of post-operative AKI for dipstick proteinuria lowered the AUC for all 4 biomarkers at the pre-operative time point and for 2 of the 4 biomarkers at the post-operative time point.

**Conclusions:**

Several factors available through urine dipstick testing are associated with increased urinary biomarker concentrations that are independent of clinical kidney injury. Future studies should explore the impact of these factors on the prognostic and diagnostic performance of these AKI biomarkers.

**Electronic supplementary material:**

The online version of this article (doi:10.1186/s12882-017-0629-z) contains supplementary material, which is available to authorized users.

## Background

Acute kidney injury (AKI) is a common and serious complication occurring post cardiac surgery. [1] It is associated with morbidity, mortality and long-term sequelae including chronic kidney disease. [2] However, serum creatinine is an imperfect marker since its levels reflect delayed functional consequences of the injury rather than direct cell injury and are not sensitive and specific in the early diagnosis of AKI. [3] For these reasons, novel biomarkers for AKI early diagnosis and prognosis are sought.

Several studies have shown the efficacy of urinary biomarkers including interleukin-18 (IL-18); plasma neutrophil gelatinase-correlated lipocalin (NGAL); kidney injury molecule-1 (KIM-1) and liver-type fatty acid-binding protein (L-FABP) to detect AKI before change in serum creatinine. [4–6] These biomarkers have the potential to improve both diagnosis and prognosis of patients with AKI.

However, non-injury related factors might impact the association between these biomarkers and AKI. It has been demonstrated previously that specimen handling and processing can affect measured biomarker levels due to differences in concentrations that depend on centrifugation speed, duration, storage temperature, storage duration, and freeze/thaw cycles. [7] Presence of hematuria and pyuria may potentially affect biomarker concentrations and assay performance in the absence of injury. We investigated the sources of variation in biomarker concentration by urine dipstick results in patients without evidence of clinical AKI.

## Methods

We examined pre- and post-operative urine specimens in the subgroup of 714 adults (from the total of 1219) who underwent cardiac surgery from six different centers from the Translational Research Investigating Biomarker Endpoints in AKI (TRIBE-AKI) a large prospective, multicenter international cohort of adult patients undergoing cardiac surgery, who did not experience post-op clinical AKI. The TRIBE-AKI cohort has been described previously. [8] We included participants with peak serum creatinine change of ≤20% from baseline and excluded patients with AKI to minimize the effect of biomarker expression due to injury. The maximum post-operative serum creatinine was based on all post-operative measurements up to the 5th post-operative day. We assayed IL-18, NGAL, KIM-1 and L-FABP in urine specimens at two time points: preoperative and first postoperative (6 h post-op). The assays for these biomarkers have been described previously. [8, 9] We examined the urine dipstick parameters for sources of variation in the 4 urinary biomarkers. We log transformed the values of these biomarkers to account for skewed distributions. We then examined the association between urinalysis findings and the concentrations of urinary biomarkers using R^2^ correlations. We also assessed the difference in log biomarker concentrations between urinalysis findings using two tailed tests of hypothesis (t-tests), since correlations may be statistically significant but there may not be a significant difference in biomarker levels between covariates. We considered a two tailed *p* value of <0.05 to be statistically significant.

Since we found proteinuria to have the strongest correlation with the urinary biomarker levels, we examined the impact of adjusting for proteinuria on the diagnostic performance of the biomarkers for stage 2 or 3 AKI in the whole cohort of 1219 participants. We calculated the center-adjusted AUC for each biomarker and additionally adjusted for urine protein as an ordinal variable on dipstick (Negative, Trace, 30+, 100++, 300+++, 2000 or more).

## Results

**Fig. 1:**
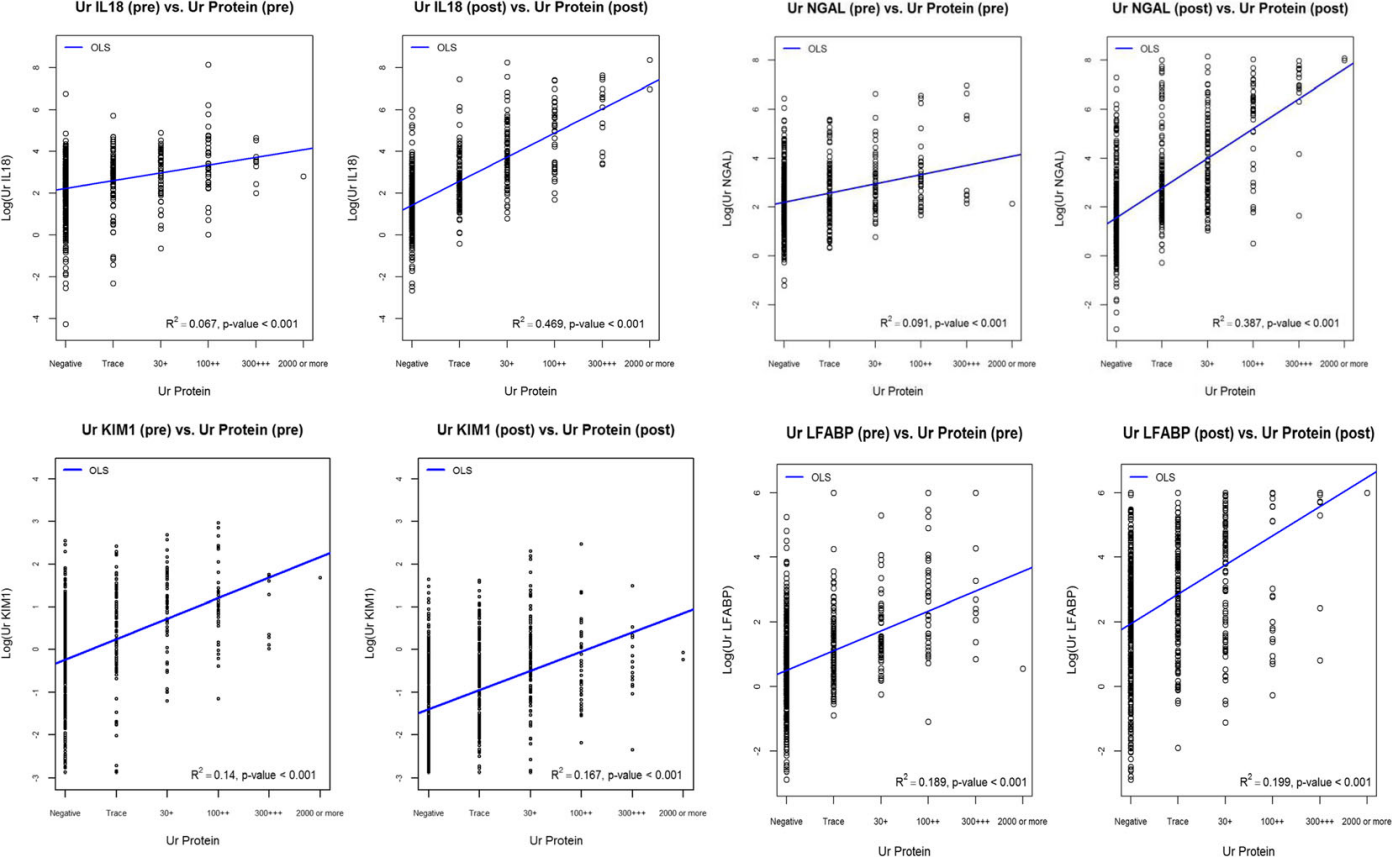
Associations of Biomarker concentrations with dipstick proteinuria. This figure demonstrates the difference in the log transformed biomarker concentrations by differing levels of dipstick proteinuria (negative; trace, ≥30 mg/mg of creatinine, ≥100 mg/mg of creatinine and ≥2000 mg/mg of creatinine). The *blue line* denotes the regression line of the biomarker concentrations vs. the dipstick proteinuria

**Fig. 2:**
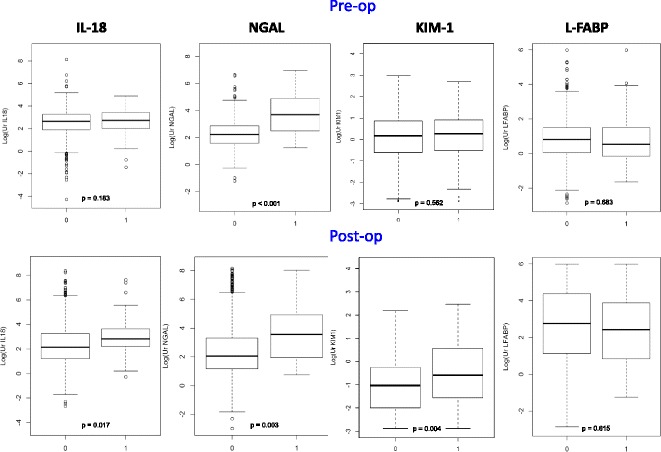
Differences in Biomarker Concentrations by Hematuria. This figure demonstrates the differences in the median and interquartile range of the log transformed biomarker concentrations by presence of hematuria. The differences are demonstrated both in the preoperative and postoperative concentrations. *P* values are from two-sample t-test allowing for unequal variances

**Fig. 3:**
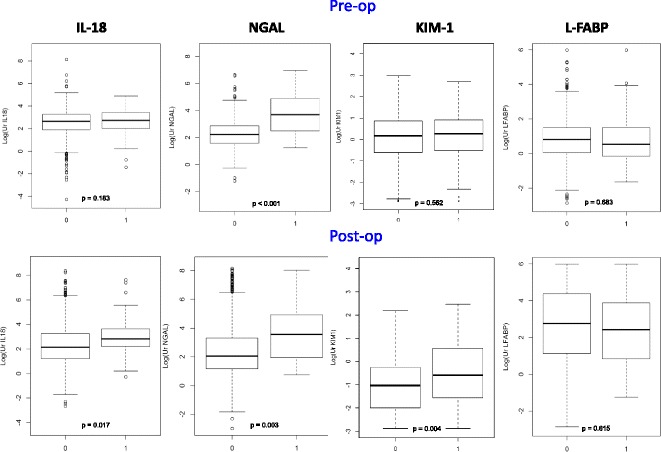
Differences in Biomarker Concentrations by Positive Urine Leukocyte Esterase. This figure demonstrates the differences in the median and interquartile range of the log transformed biomarker concentrations by presence of urine leukocyte esterase. The differences are demonstrated both in the preoperative and postoperative concentrations. *P* values are from two-sample t-test allowing for unequal variances

**Fig. 4:**
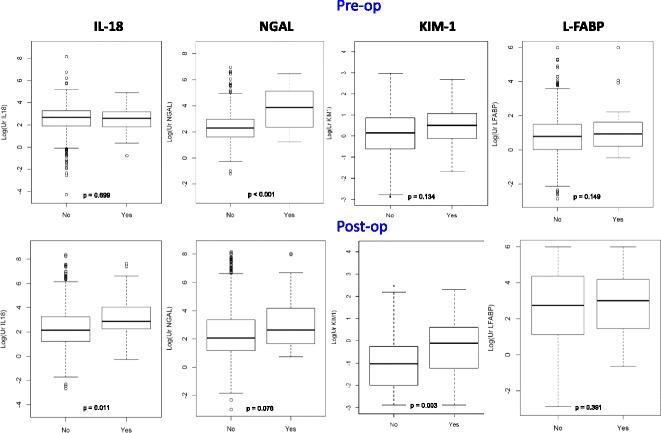
Differences in Biomarker Concentrations by Positive Urine Nitrites. This figure demonstrates the differences in the median and interquartile range of the log transformed biomarker concentrations by presence of urine nitrites. The differences are demonstrated both in the preoperative and postoperative concentrations. *P* values are from two-sample t-test allowing for unequal variances

Table 1 summarizes the baseline characteristics on the 714 TRIBE-AKI participants without evidence of clinical AKI. Table 2 demonstrates the correlations of the biomarkers with the dipstick covariates of interest. Several dipstick covariates were associated with biomarker concentrations. All 4 biomarkers were correlated with urine dipstick proteinuria pre- and postoperatively **(**Additional file 1: Table S1) The pre-operative correlations ranged between 0.067 to 0.189 (Fig. 1). Post-operative correlations were 0.167 for urine KIM-1, 0.387 for urine NGAL and 0.469 for urine IL-18. In addition, all biomarkers were correlated with hematuria, with the strongest correlation being post-operative NGAL (R^2^ = 0.21; Fig. 2). Only NGAL was correlated with leukocyte esterase pre-operatively (Fig. 3), while postoperative levels of all markers except L-FABP were very weakly correlated with urine leukocyte esterase. IL-18 and KIM-1 were weakly correlated with urine nitrite postoperatively and NGAL was weakly correlated with urine nitrite preoperatively (Fig. 4).

**Table 1:** Baseline Characteristics of TRIBE-AKI Subcohort without Clinical AKI (*n* = 714)

Characteristic	
Age in years, Mean (SD)	71.5 (10.0)
Male, n (%)	479 (67%)
White, n (%)	668 (94%)
Elective Surgery, n (%)	592 (83%)
Diabetes, n (%)	258 (36%)
Heart Failure, n (%)	159 (22%)
Hypertension, n (%)	555 (78%)
eGFR in ml/min, Mean (SD)	66.2 (18.6)
On CPB, n (%)	634 (93%)
CPB time, min (SD)	113.4 (44.5)
Preoperative MI, n (%)	176 (25%)

*eGFR* Estimated glomerular filtration rate; *CPB* Cardiopulmonary bypass; *MI* Myocardial Infarction; *STS* Society of Thoracic Surgery

**Table 2:** AUCs for Clinical AKI adjusted for Presence of Dipstick Proteinuria

Time point	Biomarker	Center-adjusted	Center- and proteinuria-adjusted
Preop	uIL18	0.52	0.48
	uNGAL	0.49	0.45
	uKIM1	0.57	0.52
	uLFABP	0.50	0.48
1st Postop	uIL18	0.62	0.65
	uNGAL	0.56	0.56
	uKIM1	0.70	0.61
	uLFABP	0.56	0.55

Center is treated as a nominal variable and proteinuria is treated as an ordinal variable in the following order Negative, Trace, 30+, 100++, 300+++, 2000 or more

Clinical AKI was defined as stage 2 or 3 AKI. There were 60 AKI events

We examined the impact of adjusting for dipstick proteinuria on the diagnostic performance of the 4 urinary biomarkers for stage 2 or 3 AKI in the overall cohort of 1219 participants (with and without clinical AKI). As shown in Table 2, the AUC for post-operative AKI was attenuated for all 4 urine biomarkers measured at the pre-operative time point after adjusting for proteinuria. For the 1st post-operative biomarker value, after adjusting for proteinuria, the AUC improved for IL-18, was unchanged for urine NGAL, and decreased for urine KIM-1 and urine L-FABP.

## Discussion

In this post-hoc analysis of a subcohort of a large, multicenter prospective cohort study of cardiac surgery patients without creatinine-based acute kidney injury, we did not find evidence of association between either pre- or postoperative biomarker levels and age or sex. However, there were significant differences in biomarker levels associated with urine dipstick findings, including proteinuria, hematuria, leukocyte esterase and nitrites.

All biomarkers were mildly correlated with hematuria, indicating that blood might interfere with biomarker levels or their measurement even in the absence of overt kidney injury evidenced by creatinine rise. Preexisting substances in the urine interfering with assays for novel analytes has been previously described. [10]

We found evidence that proteinuria is associated with all four biomarkers both pre- and post-operatively. Dipstick proteinuria is a marker of preexisting kidney damage and is associated with both the incidence and outcomes of acute kidney injury. [11] In addition, there is evidence that proteinuria may represent subclinical or early acute tubular necrosis, which has not yet manifested as a rise in creatinine. [12] Thus, the part of the correlation we observed between proteinuria and biomarker levels may be the association between proteinuria and subclinical kidney injury. When we adjusted for measures of discrimination of AKI for urine protein, all of the pre-op AUCs diminished and 2 of the 4 post-operative AUCs diminished. Since dipstick proteinuria is a readily available and inexpensive test, future studies should assess the impact of accounting for dipstick proteinuria in evaluating the predictive and diagnostic performance of novel biomarkers.

We also found evidence of an association between NGAL levels and leukocyte esterase/nitrites. Urinary NGAL has been previously shown to be elevated in patients with septic shock. [13] This is especially important, since critically ill patients, in whom novel biomarkers could potentially be deployed for diagnostic and prognostic purposes, have high rates of urinary tract infections or may have leukocyturia/urine nitrites secondary to urinary catheters. [14] This may lead to false positive AKI diagnosis in patients with no kidney injury but leukocyturia/urinary nitrites. Although, currently the mainstay of AKI management is supportive, if future therapies for AKI develop, misdiagnosis may expose patients with no kidney injury to unnecessary risk of a novel therapy.

Our study has several limitations. Since our study population was predominantly white, we could not assess racial differences in biomarker levels. In addition, we did not have urine sediment or histology to discern acute tubular necrosis in absence of serum creatinine rise and for correlation with biomarker levels. Thus, there may have been underlying subclinical injury that resulted in the correlations between the dipstick findings and the biomarkers levels.

## Conclusions

Urine dipsticks are a cheap, readily available, “point of care” test. We found significant interference from factors in urine measured by dipstick and the novel AKI biomarkers investigated here. Future studies should assess the interference of common urine elements on levels of biomarkers of interest and explore the impact of accounting for these elements on their prognostic/diagnostic performance.

## Additional file

Additional file 1:
**Table S1.** Correlations between Biomarkers and Urine Dipstick Characteristics. (DOCX 16 kb)

## Abbreviations

IL-18: Interleukin-18
KIM-1: Kidney Injury Molecule-1
L-FABP: L Fatty Acid Binding Protein
NGAL: Neutrophil Gelatinase Associated Lipocalin
TRIBE-AKI: Translational Research Investigating Biomarker Endpoints in AKI
