# Supplementary material for: Urinalysis findings and urinary kidney injury biomarker concentrations
Source: BMC Nephrol. 2017 Jul 6;18:218. doi: 10.1186/s12882-017-0629-z (PMC5499057; doi:10.1186/s12882-017-0629-z)
Supplement: Additional file 1: — Table S1. Correlations between Biomarkers and Urine Dipstick Characteristics. (DOCX 16 kb) [file 12882_2017_629_MOESM1_ESM.docx]

**Table S1.** Correlations between Biomarkers and Urine Dipstick Characteristics

| Variable | Biomarker | Preoperative | | 1^st^ Postoperative | |
| --- | --- | --- | --- | --- | --- |
|  |  | **R^2^** | **P** | **R^2^** | **P** |
| Urine Protein | **IL18** | **0.113** | **<0.001** | **0.323** | **< 0.001** |
|  | **NGAL** | **0.15** | **<0.001** | **0.292** | **< 0.001** |
|  | **KIM1** | **0.178** | **<0.001** | **0.225** | **< 0.001** |
|  | **LFABP** | **0.194** | **<0.001** | **0.122** | **< 0.001** |
| Urine Leukocyte Esterase | **IL18** | 0.003 | 0.183 | **0.008** | **0.017** |
|  | **NGAL** | **0.158** | **< 0.001** | **0.013** | **0.003** |
|  | **KIM1** | 0.001 | 0.562 | **0.012** | **0.004** |
|  | **LFABP** | < 0.001 | 0.683 | <0.001 | 0.615 |
| Hematuria | **IL18** | **0.02** | **<0.001** | **0.134** | **<0.001** |
|  | **NGAL** | **0.044** | **<0.001** | **0.205** | **<0.001** |
|  | **KIM1** | **0.026** | **<0.001** | **0.029** | **<0.001** |
|  | **LFABP** | **0.017** | **0.001** | **0.134** | **<0.001** |
| Urine Nitrites | **IL18** | 0.001 | 0.699 | **0.01** | **0.011** |
|  | **NGAL** | **0.066** | **< 0.001** | 0.005 | 0.076 |
|  | **KIM1** | 0.003 | 0.134 | **0.013** | **0.003** |
|  | **LFABP** | 0.003 | 0.149 | 0.001 | 0.391 |
